# Organic nanoparticle-doped microdroplets as dual-modality contrast agents for ultrasound microvascular flow and photoacoustic imaging

**DOI:** 10.1038/s41598-020-72795-w

**Published:** 2020-10-12

**Authors:** Yu Xu, Guoyun Sun, Eshu Middha, Yu-Hang Liu, Kim Chuan Chan, Bin Liu, Chia-Hung Chen, Nitish V. Thakor

**Affiliations:** 1 Department of Biomedical Engineering, National University of Singapore, 4 Engineering Drive 3, Singapore, 117583 Singapore; 2 SINAPSE Laboratory, 28 Medical Drive, Singapore, 117456 Singapore; 3 Department of Chemical and Bio-Molecular Engineering, National University of Singapore, 4 Engineering Drive 4, Singapore, 117585 Singapore; 4 Institute for Health Innovation and Technology (iHealthtech), National University of Singapore (NUS), MD6, 14 Medical Drive #14-01, Singapore, 117599 Singapore; 5 Department of Biomedical Engineering, City University of Hong Kong, 83 Tat Chee Avenue, Kowloon Tong, Hong Kong; 6 Department of Biomedical Engineering, Johns Hopkins University, Baltimore, MD 21205 USA

**Keywords:** Biotechnology, Engineering, Optics and photonics

## Abstract

Tumor blood vessels are chaotic and abundantly distributed, owing to their heterogeneity. Therefore, imaging techniques which reveal abnormalities of tumor vasculature play significant roles in both mechanistic and clinical diagnostic tumor studies. Photoacoustic (PA) imaging uses the intrinsic characteristics of hemoglobin, to acquire tumor hemodynamic information, while ultrasound (US) imaging provides information about tumoral vessel structures and blood flow. To improve the imaging contrast performance, hydrogel-based microdroplets were designed for both US blood flow and PA imaging in this study. The microdroplets served as carriers for PA contrast agent solution in the innermost part while oil and hydrogel formed the inner and outer layers of the droplets. In vitro experiments firstly demonstrated the dual modality contrast effects of the microdroplets on US flow determination and PA imaging. In vivo experiments were then carried out in both healthy nude mice and nude mice with subcutaneous tumor to validate the contrast effects and to monitor the duration of contrast effects in animals. Using the dual-modality microdroplets, we were able to obtain distinct edges of tumor and blood flow mapping of the tumor microvascular with improved sensitivity up to 11.09 dB for PA and 6.69 dB for US flow. Besides, the in vivo evaluation with microdroplets showed US flow enhancement for more than 60 min. Therefore, the microdroplets are able to provide the contrast effects for both US flow and PA in a relative long duration and have potential to be applied in the tumor related diagnoses and studies.

## Introduction

Photoacoustic imaging (PAI) has gained attentions over the past decades since it provides high ratio of imaging depth over spatial resolution (> 200), while the imaging scales range from organelles to organs^1^. It could reveal functional information such as cerebral blood volume (CBV), oxygen saturation, etc. PAI is initiated by the absorption of the short pulse (normally ns) laser beam. The heat generation due to the light absorption leads to thermoelastic expansion of chromophores. Subsequently, the resulting acoustic waves are detected by the ultrasound (US) transducer. Among the principles of PA generation, thermoelastic expansion is the only safe method but also the least efficient mechanism to produce PA signals on biotissues^2,3^. Besides, intrinsic contrast agent activity is evident only at certain wavelengths (e.g. the absorption coefficient of hemoglobin at near infrared (NIR) range is at least one order less compared to that at 400 nm)^4^. In addition, wavelengths in the NIR spectrum are normally chosen to increase the penetration depth of light. Therefore, exogenous PA contrast agents are necessary to enhance the sensitivity of PAI by absorbing more light. Optical absorbers such as organic dyes, metallic and organic nanoparticles are generally applied^5–7^. The absorption peaks are tunable by changing the morphologies of the nanoparticles^8^. With the application of exogenous contrast agents, PAI is able to acquire more functional and metabolism information in the region of interest (ROI).

Ultrasound imaging, on the other hand, is one of the most popular clinical imaging modalities for diagnosis, treatment and therapy^9^. The contrast agents of US work under different principles from the contrast agents of PA. Due to their highly scattering and nonlinear harmonic acoustic properties, microbubbles can enhance the differentiation of blood vessels with respect to the surrounding tissues. Thus, microbubbles are commonly applied as contrast agents in US imaging^10^. Commercially available microbubbles are typically composed of lipids, albumin and polymers. They have been extensively applied in biomedical fields, such as breast imaging, US-triggered drug delivery, vascular remodeling^10^, mapping blood flow in hearts, livers and kidneys^6^, and localized US microscopy^11^. Although PA system could visualize the microvasculature over skin^12^, brain and monitoring the formation of angiogenesis around tumor in real-time scale^13^, US imaging could still supplement certain information, such as US blood flow in research studies and clinical applications. Since both of them use ultrasonic detection, PA and US have been increasingly integrated in terms of hardware instrumentation to provide simultaneous functional (PA) and structural (US) information of the targeted tissue areas. This dual-modality approach to imaging has also led to the need for contrast agents for both imaging modalities.

Several related researches have been conducted in the last decade. The dual-modality contrast effects could be achieved by simply applying porphyrin-phospholipid (Porshe) conjugated microbubbles (MBs). The Porshe MBs have resonance peak about 9–10 MHz for US and optical absorption peak at 700 nm^10^. However, they are not optically tunable for the PA contrast effect and even at 700 nm, the PA signal of the Porshe MBs is only slightly bigger than the intrinsic PA signal from blood^14^. Previous studies have also investigated combining microbubbles with optical absorbers, such as nanoparticles and organic dyes^6,7,9,14–16^. Some of them utilized liquid perfluorocarbons (PFCs) loaded nanodroplets as the carriers of optical absorbers, where PFCs provided triggerable platforms for US imaging by converting nanodroplets into microbubbles through vaporization^14–16^. In these studies, droplet vaporization was induced by optical irradiation instead of acoustic methods, which allowed the activation of droplets at locations where acoustic waves could not penetrate under FDA safety limit strengths^14,16,17^. The drawback of using PFC as the platform is quite straightforward. Although the expansion ratio of PFC is approximately 5.5, the size of the triggered microbubbles varies over large scales and is not readily controlled. For example, in Ref.^12^, 200 nm PFC loaded nanodroplets were mostly vaporized into microbubbles distributed from 1 to 6 μm, while in Ref.^15^, with the same size nanodroplets, the diameter of the largest microbubble was found to be close to 50 μm after vaporization. Besides these previous methods, microbubbles have also been generated in methylene blue (a PA lymph node tracer in breast cancer) solution by sonicated activation or using microfluidics to perform as dual-modality contrast agents^6,18^.

The disadvantage of using microbubbles as US contrast agents is quite evident: the circulation time of commercial microbubbles is short. The elimination half-life time is 6 min for microbubbles from Sonovue (Bracco Spa, Milan, Italy), while for Albunex (Molecular Biosystems, San Diego, CA, USA), after 3 min, 70% of the microbubbles in the bloodstream are cleared^19^. Such a short circulation time is not enough for the experiment needing long durations of observation, such as monitoring the response to anti-angiogenesis therapy to restore blood flow^20^. Therefore, in clinical practice, multiple dosages are required during one diagnostic imaging session. Besides, the heat absorption due to laser irradiation increases the gas diffusion rate of microbubble based dual-modality contrast agents, which shortens the circulation time even more.

In order to develop a new structure for the dual-modality contrast purpose with long-circulation time, hydrogels, which were firstly developed as biomaterials for human use and have been applied in a large number of studies of long-term drug delivery^21,22^, were selected as the manufacturing material. To take advantage of their unique properties of biocompatible and biodegradable, we synthesized hydrogel-based droplets within microfluidic chips as the sources of US contrast effect and also as carriers of PA contrast agents. Different from previous studies, during the manufacturing of the hydrogel-based contrast agents, we replaced metallic nanoparticles and organic dyes with conjugated polymer (CP) nanoparticles (NPs), which is one type of the semiconducting polymer nanoparticles (SPNs) as photo-absorbers, to take advantage of their high absorption coefficients, controllable dimensions, organic and biologically inert materials and easily functionalized surface^23^.

In this paper, an organic nanoparticle doped, hydrogel-based multiplayer structure is demonstrated as dual-modality contrast agent for both US blood flow and PA imaging and is shown to achieve the desired long circulation time. To the best of our knowledge, this is the first time that polymer-based nanoparticles and hydrogel are used as dual-modality contrast agents for US flow and PA imaging. And it is also the first time that investigation of long circulation time for dual-modality contrast agents has been demonstrated. Our demonstration and verification of the contrast effects include the manufacturing process, in vitro studies in transparent tubes and in vivo studies in both healthy and tumor induced nude mice. The results also show the potential application of slow drug release of our nanoparticle-hydrogel formulation.

## Results

### Microdroplets evaluation

**Figure 1:**
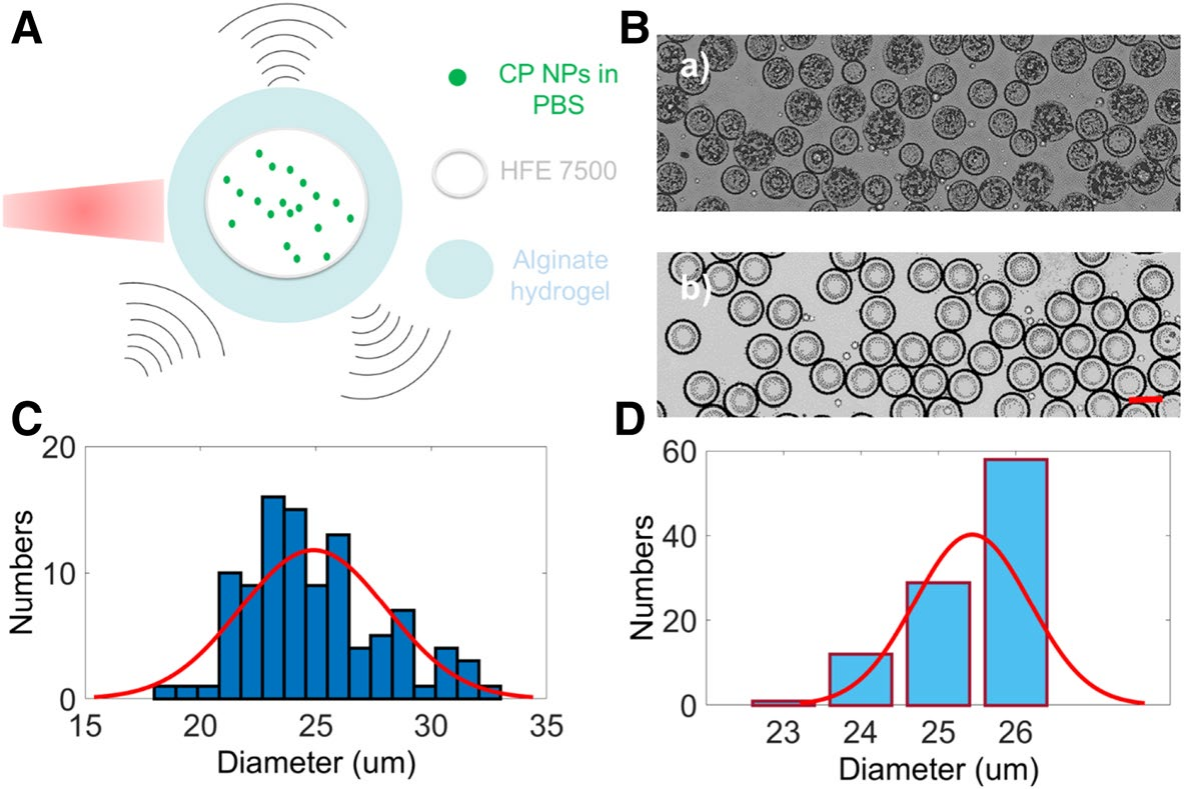
Schematic diagram and size distributions of the microdroplets: (**A**) The structure of the microdroplets consists of three layers: alginate hydrogel (aqueous phase)—HFE 7500 (oil phase)—CP NPs in PBS solution (aqueous phase) from outside to inside. (**B**) (a,b) are the images of microdroplets manufactured with and without surfactants, respectively. The scale bar is 25 μm. (**C**) Size distribution of the microdroplets with surfactants. The mean value of the normal distribution fit is 24.9 μm. (**D**) Size distribution of the microdroplets without surfactants. It has a mean value of 25.6 μm although is not normally distributed.

Microdroplets manufacturing in the “Materials and methods” section provides the manufacturing details. After manufacturing the microdroplets, we firstly evaluated the size distribution and encapsulation efficiency. The structure of the microdroplets is shown in Fig. 1A. The nanoparticle solution was sealed by HFE 7500 (Sigma, St. Louis, Missouri, USA), and then enclosed by alginate hydrogel. The PA intensity was enhanced since the absorption of laser light was increased by the nanoparticle inside the microdroplets. US intensity, on the other hand, is enhanced by the backscattering from the multilayer structure because of the mismatched densities (Density: HFE 7500 > alginate hydrogel > nanoparticle solution). Figure 1B (a) gives us the picture of the microdroplets taken by a live cell inverted microscopy (DMI8, Leica, Wetzlar, Germany) before crosslinking and washing off the outermost oil layer. 100 droplets were randomly chosen from the picture to calculate the size variations, and the size was normally distributed with the mean value of 24.9 μm, as shown in Fig. 1C. The results also showed that the size varied in a wide range from 18 to 33 μm while most of the droplets are concentrated in between 21 to 29 μm. The wide range of size distribution is highly likely due to the large viscosity of the surfactants. To verify the effects, the surfactants (i.e. alpha-tocopherol (Sigma, St. Louis, Missouri, USA) and tween 80 (Sigma, St. Louis, Missouri, USA) in our case) were removed when another group of microdroplets were made following the same procedure. Once again, 100 microdroplets were chosen from Fig. 1B (b), which is the picture of the microdroplets without surfactants, to calculate the size distribution. The results are shown in Fig. 1D. More than half of the microdroplets are approximately 26 μm and the sizes are narrowly distributed between 23 to 26 μm. The mean value of the size distribution of Fig. 1D is 25.6 µm. Although the surfactants affect the uniformity of the microdroplets, without adding them the yield of oil droplet enclosed with nanoparticle solution was too low, compared between Fig. 1B (a, b). Thus without adding surfactants, the microdroplets could not be used as the dual-modality contrast agents.

After manufacturing, the final products (microdroplets) were collected and dissolved in PBS. 10 μl of the microdroplets was extracted and further diluted with a ratio of 1:19 using PBS. After dilution, 10 μl of the solution was extracted and visualized under an inverted microscope (Eclipse Ti, Nikon, Melville, U.S.A) using C-chip (NanoEntek Inc, South Korea). The amounts of the encapsulated and vacant microdroplets were counted. By calculating the ratio of encapsulated ones over the number of total microdroplets, the encapsulation efficiency was found to be 69% (Supplementary Information 3. Microscopic image of the microdroplets and microdroplets in C-chip. Supplementary Fig. S2). The biodegrability of the microdroplets was also verified to ensure the microdroplets would degrade after their usage in animal bodies. The pH value of the microdroplets solution was changed from acidic (since acetic acid was added during the manufacturing process, shown in “Materials and methods”, Microdroplets manufacturing) to neutral (same as animal blood). The microdroplets were then monitored under the inverted microscope using C-chip over 24 h time scales. The biodegrability of the microdroplets was demonstrated since most of them were eliminated after 24 h (Supplementary Information 4. Biodegradability verification. Supplementary Fig. S3).

### In vitro testing

**Figure 2:**
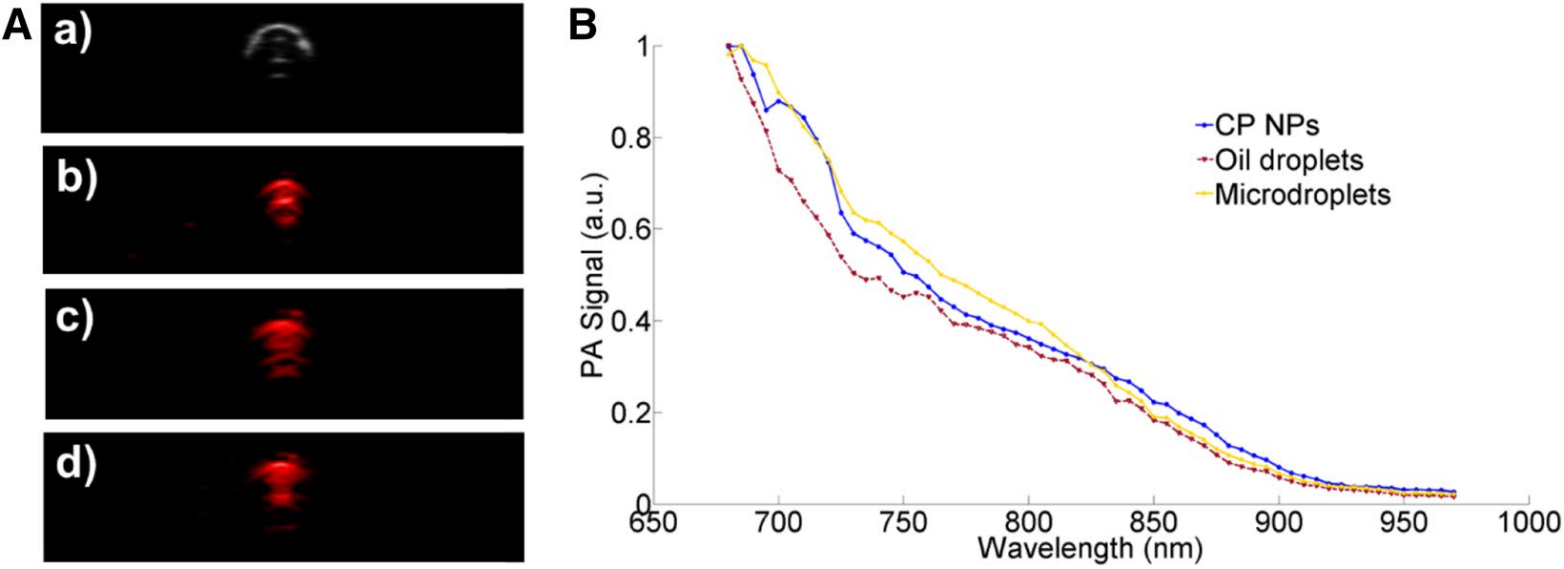
PA images and spectrums of three samples: (**A**) Imaging results of samples in test-tubes: (a) The ultrasound image of the tube filled with CP NPs. (b–d) are the PA images of CP NPs, oil droplets and hydrogel microdroplets with CP NPs inside, respectively. The illumination wavelength was 750 nm. (**B**) The normalized PA spectra of these three samples. There is no significant change in the spectra when CP NPs are covered by both oil and hydrogel.

The influence of the external material (HFE 7500 oil and hydrogel) on the PA spectrum of CP NPs was investigated at first. Figure 2A (a) indicates the B-scan ultrasound image of the sample filled tube under a linear transducer array with the central frequency of 40 MHz. (b–d) are the PA images of CP NPs, oil droplets and microdroplets at 750 nm. The oil droplets (products of step 2 of Microdroplets manufacturing in “Materials and methods” section) are PBS solution of CP NPs covered with HFE 7500. The experimental conditions and system setup were kept identical for the same evaluations in this study. To acquire PA spectra, the laser wavelengths were swept from 680 to 970 nm with a step size of 5 nm. After the data were collected, the PA amplitudes were normalized in the same graph to compare the patterns of PA spectra of these three samples as shown in Fig. 2B. It turns out that although there is a gap between the CP NPs and oil droplets in the 700–830 nm wavelength range, due to the enclosure of hydrogel, the PA spectra of CP NPs and microdroplets display similar trends.

Subsequently, the contrast effects on US flow and PA imaging were investigated in transparent tubes. The US flow modality of our imaging system is based on Doppler imaging. In Doppler imaging, Doppler shift frequency is the primary data to be measured. According to the Doppler equation:

1 $$ {\text{Doppler}}\,{\text{shift}}\,{\text{frequency}} = { }\left( {2 \times {\text{f}}_{o} \times {\text{v}}_{r} \times {\cos}\vartheta } \right)/v_{s} {.} $$

In the above equation, f_o_ is the operating frequency of the ultrasound probe. v_r_ is the velocity of the reflector (red blood cells, microbubbles, microdroplets in our case). θ is the angle between the ultrasound beam and the flow direction. v_s_ is the speed of sound.

**Figure 3:**
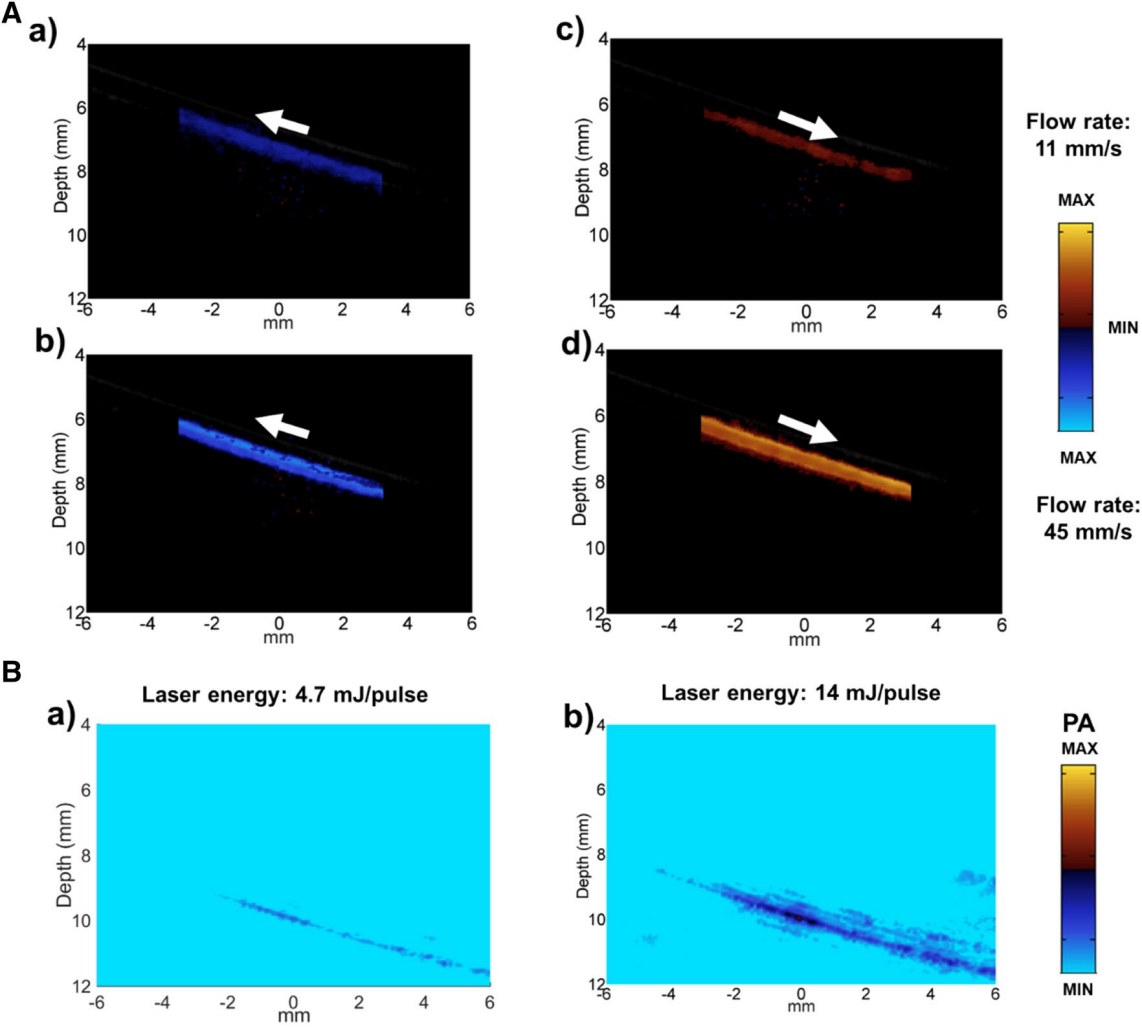
US flow and PA images of the microdroplets in transparent tubes: (**A**) US flow imaging with flow speed of 11 mm/s (a,c) and 45 mm/s (b,d). The white arrow indicates the flow direction (**B**) PA images with laser energy 4.7 mJ/pulse (subfigure a)) and 14 mJ/pulse (subfigure b)). The irradiation wavelength is 750 nm.

In our experiments, f_o_ was kept the same since we are using the same probe. v_s_ is constant in the same medium. To keep the velocity of reflector v_r_ as the only variable, θ needed to be fixed. Thus the position of the tube was fixed in water tank and the tube was tilted 25° with respect to the x–y plane. The diluted microdroplets were extracted into a 1 ml syringe and then pumped by PHD2000 (Harvard Apparatus, Holliston, Massachusetts, USA) syringe pump with constant speed. The flow directions and flow speeds were all controlled by the syringe pump. Figure 3A represents flow imaging with flow speed of 11 mm/s [i.e. (a) and (c)] and 45 mm/s [i.e. (b) and (d)]. (a) and (b) are the flow images when we infused the syringe. (c) and (d) are the flow images when we withdrew the syringe. The white arrow indicates the flow direction of the microdroplets, which is marked as color blue and orange too. Note that the intensity of the flow imaging is determined by both the strength of contrast effects and the flow speed of the microdroplets. Thus, to compare the contrast effect on US flow, the flow velocity needs to be kept identical. Figure 3B shows the PA images of the microdroplets in tubes. The PA effects were activated by the laser pulse at 750 nm, which is the absorption peak of CP NPs^24^. The irradiation energy is 4.7 mJ/pulse for subfigure a) and 14 mJ/pulse for subfigure (b). Larger laser energy contributed higher PA contrast and the PA contrast happened at the same position in both PA images, thus validates the PA contrast effects of the microdroplets. Overall, Fig. 3 validates the capability of the microdroplets to enhance both US flow and PA imaging.

**Figure 4:**
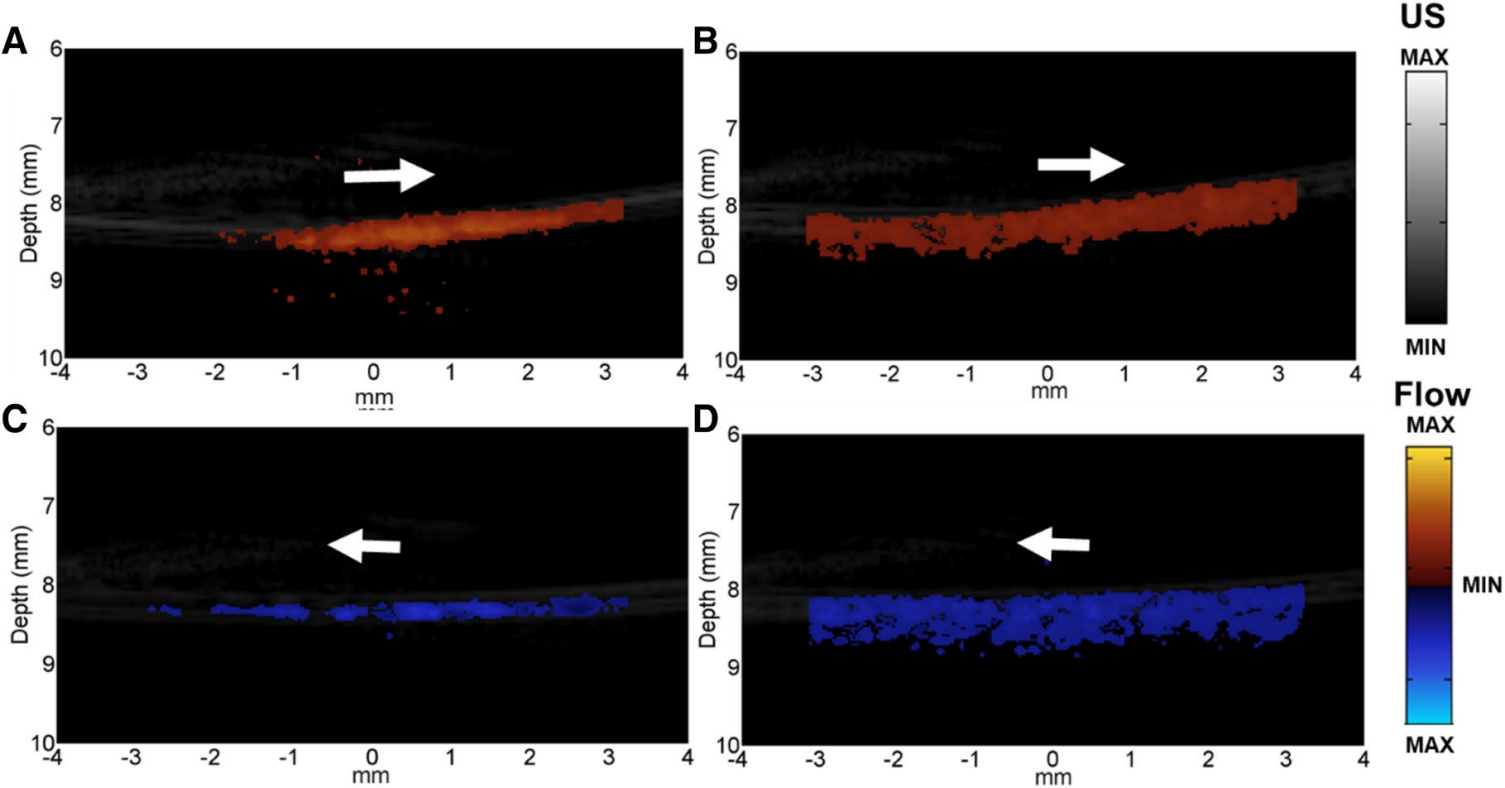
Comparison of the contrast effects of US flow between microbubbles (Sonovue™) and microdroplets: (**A**) and (**C**) are flow images of microbubbles (Sonovue™). (**B**) and (**D**) are the flow images of microdroplets. The white arrow indicates the flow directions.

To compare the contrast effects of the hydrogel-based microdroplets with commercial microbubbles on US flow imaging, Sonovue™, which is commonly clinically used, was employed as control for the comparisons (the samples were prepared following the instructions provided by the manufacturer). After preparation of the materials, both microbubbles and microdroplets were extracted into the syringe and then pumped by PHD 2000 syringe pump with the same directions and speed (11 mm/s). The yield of the microdroplets was 1/50th compared with that of microbubbles. As indicated in Fig. 4A,B, the microbubbles and microdroplets were withdrawn first. The intensity of the US flow image due to microbubbles was almost three times of the intensity of the US flow image from microdroplets, which means the sensitivity of the microbubbles in Fig. 4A was three times as the sensitivity of the microdroplets. However, the microdroplets gave us a larger scale of US flow imaging which revealed more information of the outside structure. After that, the microbubbles and microdroplets were infused with the same speed (11 mm/s), as shown in Fig. 4C,D. This time, the magnitude of the enhancement on US flow imaging was almost the same between microbubbles and microdroplets. In addition, comparing the area of the enhancement, the infused microbubbles contributed much less effective area (Fig. 4C) compared with withdrawn microbubbles (Fig. 4A). On the contrary, the area of the enhancement for microdroplets were almost the same between infused (Fig. 4D) and withdrawn (Fig. 4B) movements. The differences of the areas of the enhancement exhibited two properties of the microbubbles. First of all, the microbubbles are more fragile and easily broken compared with microdroplets. Secondly, the circulation time of the microbubbles is much shorter compared with microdroplets. Since all the movements were under the same speed, low contrasts of the images (Fig. 4B–D) are due to low enhancements from the contrast agents.

### In vivo experiment

Before conducting the in vivo experiments, the concentration of the microdroplets solution was diluted to $3.8\times {10}^{6}$/mL, which is three orders of magnitude less than the yield of the commercial microbubbles^6^. The pH value of the solution was changed to approximately 7.4 as the pH value of blood is regulated between 7.35 to 7.45. Although their biodegradability has been verified (Supplementary Information 4. Biodegradibiltiy verification and Supplementary Fig. S3), the microdroplets were further tested in vivo in healthy animals by injecting via tail vein and keeping the animals under consistent monitoring for over 15 min to ensure the safety of the three layer structures. The cell toxicity and animal experiments of the CP NPs have been conducted by the previous researches^24,25^, and thus were not repeated.

**Figure 5:**
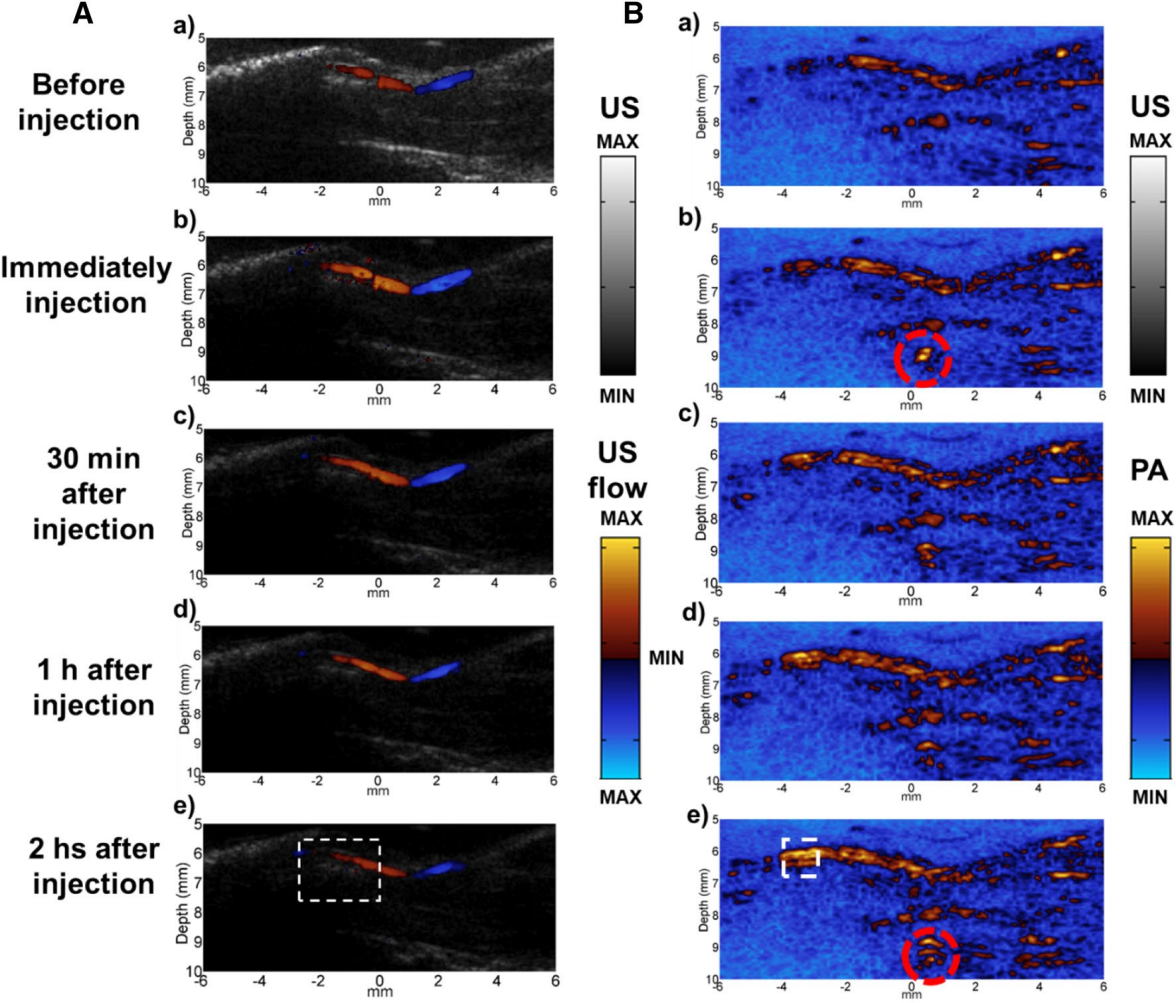
The images of US (blood) flow (**A)** and PA (**B**) at different time points: Both subfigure a) in (**A**) and (**B**) were the US flow and PA image before injection. (b–e) were the US flow and PA images immediately after injection, 30 min after injection, 1 h after injection and 2 h after injection respectively. White dashed squares are the area evaluated quantitatively.

Our in vivo studies were conducted in two steps. In the first step, a large femoral vein in a healthy animal (the animal preparation is shown in Supplementary Fig. S4) was imaged with US flow and PA imaging modalities at different time points (Fig. 5A,B). The animal was put on a tunable animal stage where the femoral vein was facing upwards. Agarose gel with 5 mm thickness was put on top of the imaging area with ultrasound gel applied on both sides. There were three reasons for the application of agarose and ultrasound gel. First of all, the solidified agarose could flatten the surface of the bio-tissue to decrease the artifacts from probe movement. Secondly, the system provides the best imaging quality if the region of interest (ROI) locates at the focal area of the PA system (approximately 10 mm for our system). The agarose gel was applied so that the superficial area of the animal, such as femoral vein in this case, could locate at the focal region of the system. Last but not the least, the application of solidified agarose and ultrasound gel was to couple the PA and US flow signals to reduce the signal loss. The same reasons applied for the tumor induced animal study as well.

In order to verify the properties of long circulation time and contrast effects of the microdroplets, the US flow and PA images were taken before the injection of the microdroplets, immediately after the injection, half an hour, one hour and two hours after the injection, as shown in Fig. 5A,B respectively. The US flow provided by the microdroplets showed largest enhancement immediately after the injection and then decayed over time, as shown in Fig. 5A. The PA enhancement, on the other hand, had been increasing since the injection of the microdroplets and reached the maximum contrast at the last time point (two hours after injection), as shown in Fig. 5B. The enhancement of the PA not only showed a clearer boundary of the vascular structure at the surface, but also exposed deeper PA targets (i.e. in the red dashed circles in Fig. 5B) that was not reached by this modality before. The data in the dashed squares was evaluated quantitatively and will be covered in Fig. 57.

In the second step of the in vivo studies, nude mouse with subcutaneous tumor was employed to illustrate how the hydrogel based dual-modality contrast agents achieved long term enhancement, especially in US flow imaging, and revealed more information about the vasculature system around the tumors. A nude mouse with 1.2 cm subcutaneous tumor was selected for this study. Agarose gel with 5 mm thickness was put on top of the tumor with ultrasound gel applied on both sides too.

**Figure 6:**
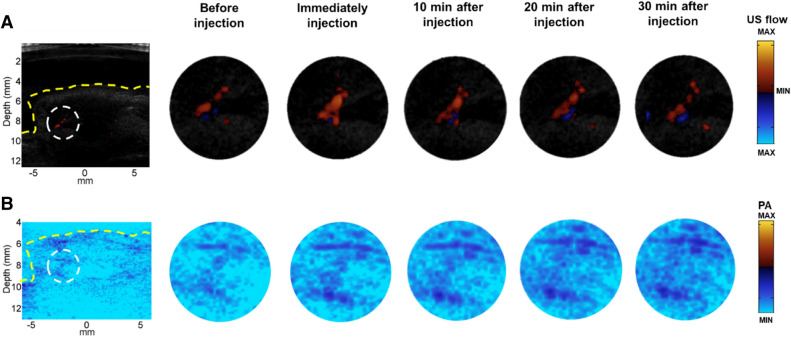
US flow (**A**) and PA (**B**) images of the subcutaneous tumor. The yellow dashed curves indicate the outline of the tumor. White dashed circles are the areas evaluated quantitatively.

Firstly, the US flow and PA images of the subcutaneous tumor over different time points are presented in Fig. 6. The yellow dashed curves indicate the outline of the tumor. The images were taken at five different time points: before injection, immediately after the injection, 10 min, 20 min and 30 min after the injection of the microdroplets. After the injection of the microdroplets, the contrast effects of both US flow and PA improved. The results were the same as the results of the healthy animal; the US flow enhancement provided by the microdroplets decayed over time and the PA enhancement kept rising. The contrast effects of the areas in the white dashed circles (marked in Fig. 6A,B before injection) were evaluated quantitatively.

**Figure 7:**
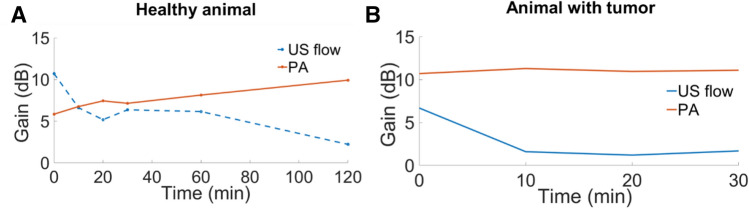
Enhancement of US flow and PA for healthy animal (**A**) and animal with tumor (**B**). For healthy animal (**A**), the enhancement of the contrast was evaluated at time points of immediately after the injection, 10 min, 20 min, 30 min, 1 h and 2 hs after the injection of the microdroplets. For animal with subcutaneous tumor (**B**), the enhancement of the contrast was evaluated at time points of immediately after the injection, 10 min, 20 min and 30 min after the injection of the microdroplets.

Quantitative comparison was done by analyzing the enhancement in the dashed box area in Fig. 5 and dashed circle area in Fig. 6. Not only the enhancement of the contrast of each pixel was considered, but also the increment of the enhanced areas (i.e. number of enlightened pixels) was calculated in as the contrast effects of the microdroplets. Figure 7A illustrates the enhancement results of the healthy animal from Fig. 5. Besides the time points presented in Fig. 5, 10 min and 20 min after the injection of the microdroplets were included for the quantitative evaluation too. The results showed that the US flow imaging was mostly enhanced immediately after the injection and the intensity decreased over time (enhancement of the imaging strength decreased from 10.71 to 2.22 dB after 2 h). The enhancement of the US flow imaging not only gave us better sensitivity to imaging, but also revealed some structures that did not display before injection. While for PA, the contrast effects increased as time went on (enhancement increased from 5.85 to 9.93 dB after 2 h). At two hours after the injection, the microdroplets contributed least US flow contrast and most PA enhancement. The enhancement results of the animal with tumor are shown in Fig. 7B. Same as the healthy animal, the microdroplets provided the largest US flow enhancement immediately after the injection and the contrast effect decayed over time (changed from 6.69 to 1.69 dB after 30 min). PA enhancement was kept a high value after the injection and increased a small portion over time (10.70–11.09 dB after 30 min).

**Figure 8:**
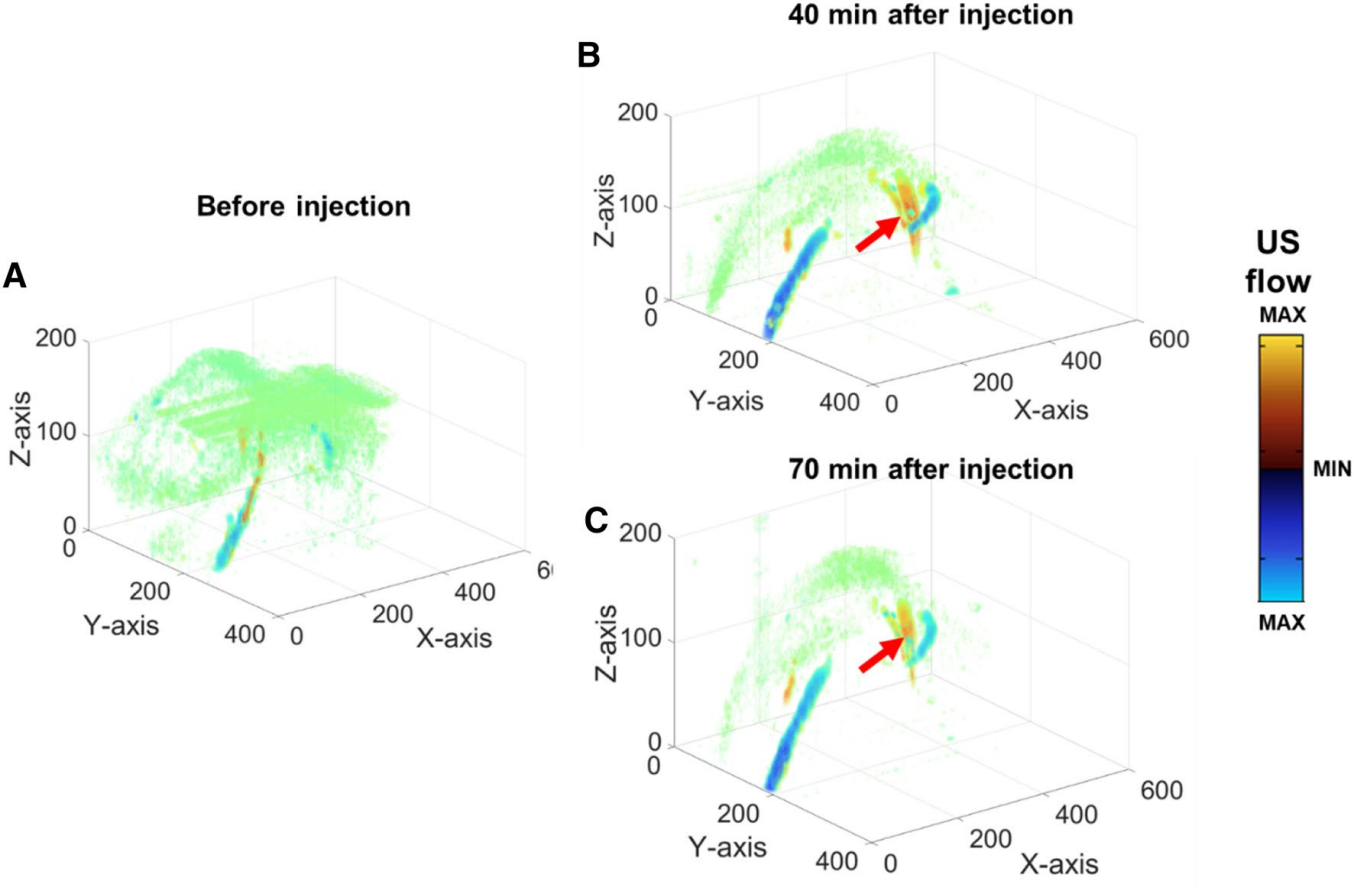
3D US flow images of the subcutaneous tumor. The blue and orange colored structures were the blood vessels inside the subcutaneous tumor. The green shadows were the noises created by the scanning stage movement. The vessels structures pointed by the red arrow were only displayed after the injection of the microdroplets. Since the microdroplets are able to reveal more details of the vascular structures and the angiogenesis process highly relates to the diagnosis and treatment of tumor, these dual-modality contrast agents can be applied in the related studies.

To further elaborate the contrast effect of the microdroplets on US flow in tumor induced nude mouse, 3D US flow images of the same tumor induced animal presented in Fig. 6 were taken before the injection of the microdroplets (Fig. 8A), 40 min (Fig. 8B) and 70 min (Fig. 8C) after the injection of the microdroplets. Based on the resolution of the US probe (i.e. approximately 150 µm^26^), we took 50 um as the step size. The number indicated on X, Y and Z-axis in Fig. 8 was the amount of the step size. In Fig. 8A–C, the blood vessels were presented in the colors of blue and orange, and the green shadow was the noise due the movement of the scanning stage. Compared with the 3D image before the injection, both Fig. 8b,C showed enhanced contrast effect on US flow. They also revealed structures (e.g. pointed by the red arrows) that weren’t showed before the injection. Thus the enhancement of the US flow due to the microdroplets could at least be effective for more than one hour.

## Discussion

The Doppler signal comes from the US scattering when acoustic waves reach the interfaces of the targets. The reactions of the US waves can be categorized into three types. If the dimensions of the sub-structure interface are much larger than the acoustic wavelength, reflection occurs. If the dimensions equal to the size of the wavelength, the acoustic waves scatter in some but not all directions. This is also referred to as non-specular reflection. If the dimensions are much smaller than the wavelength (i.e. red blood cells and microbubbles), Rayleigh scattering occurs, under which the US waves scatter in all directions. Therefore, the signals for the Doppler assessment of the intrinsic US blood flow mainly come from the Rayleigh scattering. In our studies, we want to utilize the non-specular scattering in which the size of the interface is close to the wavelength. The bandwidth of the transducer array we used for US flow imaging ranges from 6 to 30 MHz. Considering the speed of sound in bio-tissues, the acoustic wavelength is roughly 50 μm. Since the size of the output channel of the microfluidic chip dominates the size of the microdroplets, in our case, we selected microfluidic chip with output microchannel diameter of 25 μm, which is close to the US wavelength and avoid blocking the blood vessels too.

In this study, the physical properties of the microdroplets, such as the size distribution and biodegrability, were tested firstly. Secondly, in vitro test was conducted to verify the contrast effects of the microdroplets on US flow and PA. The comparison with commercial microbubbles revealed comparable contrast effect on US flow and longer circulation time of the microdroplets. Finally, the in vitro test were performed in both healthy and tumor induced nude mice. Figures 5 and 6 showed contrasted enhancement for the two imaging modalities (PA and US flow), in which the enhancement for US flow decreased as time went on and the enhancement for PA kept increasing. This phenomenon can be explained by the biodegradable property of the alginate hydrogel, which led to the decomposition of the structure of the microdroplets in nude mouse. Since the US flow enhancement is produced by the backscatter of the multilayer structure, damage to the structure resulted in the attenuation of the intensity of US flow. However, the multilayer structure of the microdroplets also partially attenuated the intensity of PA generated by the inside CP NPs. Thus the enhancement of PA was small immediately after the injection and with the decomposition of the microdroplets, damping of the PA waves decreased too. As results, at the last observing time points (2 h for healthy animal and 30 min for animal with tumors), the contrast agents we injected provided the least enhancement for US flow but the most enhancement for PA. However, compared the results of Fig. 7A,B, the enhancement of US flow decayed faster in animal with tumors. This could due to the difference of the pH value. Before the in vivo experiments, NaOH was added to neutralize the pH value so that the microdroplets solution could be used with animals. Since hydrogel is sensitive to the pH value and the decomposition of the hydrogel based structure is accelerated in an alkaline environment, single drop of the NaOH solution could change the enhancement results evidently. This can also be verified by the PA enhancement results in Fig. 7, which showed a high value immediately after the injection in the tumor induced animal that was almost the same as the PA enhancement 2 hs after the injection of the microdroplets in healthy animal. Therefore, for the future studies, the structure of the microdroplets could be improved to be more robust and less sensitive to the pH value, in order to provide longer circulation time and contrast effects. Currently, the three layer structures can be used for animal studies that need higher US flow and PA contrast within one or two hours. If larger PA enhancement is necessary at the starting time point, the PA effects can be improved by using higher concentration since the yield of the CP NPs was relatively small once released in the vasculature systems.

In summary, in order to achieve dual-modality contrast effects for both US flow and PA imaging, a three layer structure with aqueous phase–oil phase–aqueous phase has been developed. After the evaluation of the physical, optical and other properties of the microdroplets, animal experiments were conducted with both healthy and tumor induced nude mice. From in vivo test, we verified that the microdroplets achieved long circulation time and served as dual-modality contrast agents. The in vivo evaluations also proved that these properties were useful in the tumor vascular imaging. Future researches should establish their utilities in the diagnosis and treatment of tumors. Besides, this is the first time that the hydrogel based structure was used for contrast agent of US flow and PA imaging. And it is also the first time that SPNs (CP NPs in our case) have been used to provide PA contrast effects in dual-modality contrast agents of US flow and PA. This dual-modality contrast agents can be further investigated in several ways. If multifunction NPs were used, such as NPs that could provide contrast effects not only for PA, but also for MRI^24^, the additional functions of NPs could also be utilized and thus the microdroplets would provide triple (e.g. US flow, PA and MRI), quadruple or even more functions in the same structures. Last but not least, considering the biodegradable property of hydrogel, this structure could also be combined with slow drug release applications in the future.

## Materials and methods

### Nanoparticle manufacturing

For effective biological applications, CP molecules need to be rendered into water dispersible nanoparticles through nanoparticle fabrication methods, which generally include spray drying, solvent displacement method (nanoprecipitation), emulsion solvent diffusion, solvent evaporation, salting-out and dialysis^27^. Among these, nanoprecipitation is one of the simpler, faster and more straightforward techniques to synthesize small size and uniform NPs. This is also known as solvent displacement based method where the polymers are dissolved in a water miscible organic solvent, and rapid mixing of solvent into anti-solvent (water) results in the spontaneous formation of nanoparticles. Nanoprecipitation through microfluidic glass capillary mixer has gained some attentions as it exhibits tunable physiochemical properties and efficient mixing^28,29^. Due to high surface area to volume interaction, microfluidic mixers provide the benefits of enhanced and uniform mixing during NP fabrication. Also, they provide precise control over the size of NPs by varying flow parameters, such as, Reynolds No (Re) and inner to outer flow or velocity ratio. The details of synthesis of CP NPs are shown in the Supplementary Fig. S1. Calculation of Reynolds number and flow velocity are shown in Supplementary Information, Part 2.

In this study, we have synthesized small and uniform CP NPs by using microfluidic glass capillary. For biological applications, CPs are processed into water-dispersible nanoparticles with 1,2-Distearoyl-sn-glycero-3-phosphoethanolamine-polyethylene glycol (DSPE-PEG_2000_) as the matrix through the process of nanoprecipitation. Same as our previous study, we have fabricated microfluidic coaxial glass capillary mixer and synthesized CP NPs at flow rate of 34 ml/min (Re 320) (Supplementary Fig. S1 and Supplementary Equation S3)^29,30^. At Re 320, we obtained the CP NPs with an average diameter of 48 nm and polydispersity of 0.14. Based on the previous study, the absorption peak of these CP NPs is 750 nm^23^.

### Microdroplets manufacturing

The microdroplets were fabricated in four steps using polydimethylsiloxane (PDMS) microchip. First of all, CP NPs were dissolved in PBS solution with concentration of 0.5 mg/ml as the aqueous stream and HFE 7500 with 1 wt% pico-surf was served as the oil stream. The aqueous stream and oil stream were co-injected into the microchip pumped by PHD2000 (Harvard Apparatus, Holliston, Massachusetts, UAS) with flow speeds of 2 μl/min and 8 μl/min respectively. The resulting product was an oil–water structure with diameter of 10 μm (the oil droplets were tested in Fig. 2). Then the oil droplets, alginate, EDTA-Ca, alpha-tocopherol and tween 80 were mixed to form emulsions and co-injected with the same oil stream as step 1 (HFE 7500) into a flow-focusing PDMS microchip to form an oil–water–oil–water structure with diameter of approximately 24.9 μm^22^. The flow speed were 5 μl/min for emulsions and 10 μl/min for oil stream. In the third step, the microdroplets from step 2 were collected in HFE 7500 with 2 vol% acetic acid for crosslinking purposes and we let the product stand still overnight. Lastly, the outer oil phase was washed off with 1H,1H,2H,2H-Perfluoro-1-octanol (Sigma, St. Louis, Missouri, USA) to achieve a water–oil–water three layers structure. The final products were dissolved in PBS for in vitro and in vivo test later on.

### Experimental setup of US flow and PA imaging

**Figure 9:**
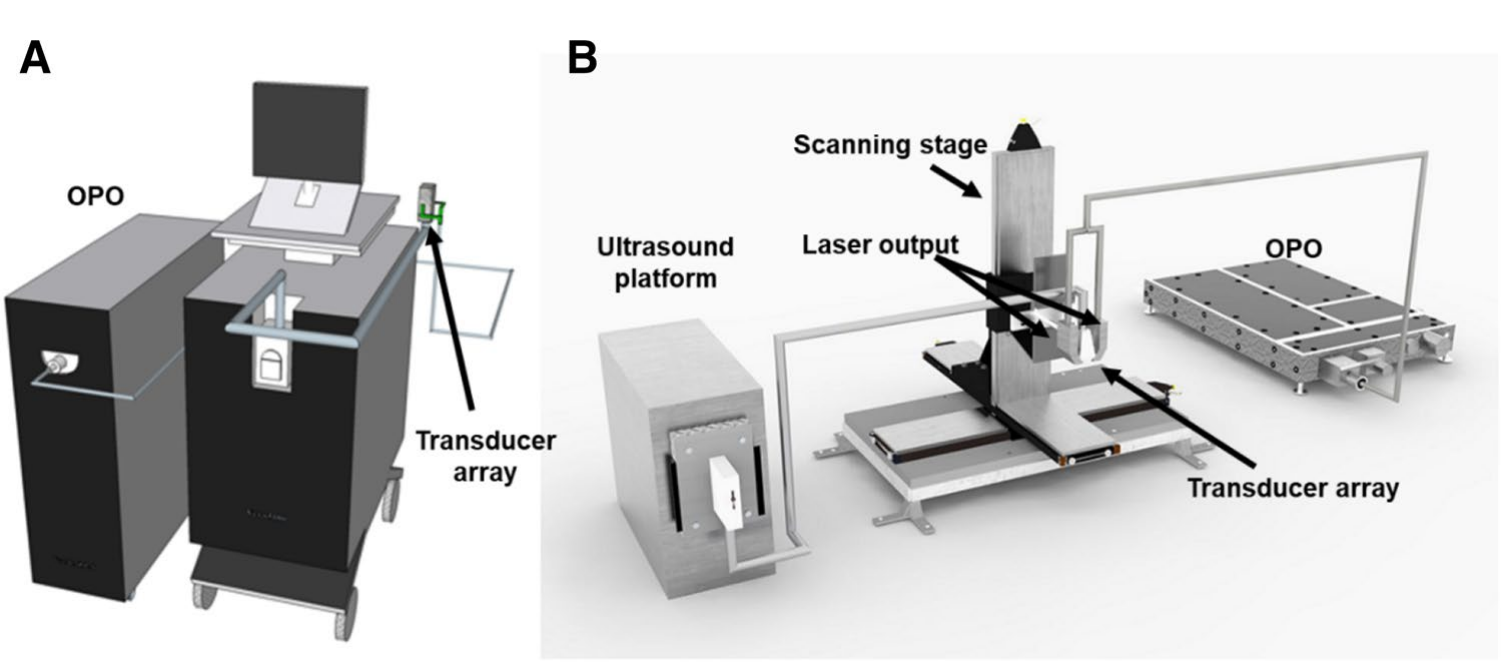
Experimental setups for the imaging systems: (**A**) Commercial PA system (Vevo LAZR-X) equipped with ultrasound detector MX550D, of which the central frequency is 40 MHz. (**B**) Self-developed handheld photoacoustic real-time imaging system. The system includes a pulsed laser with repetition rate of 100 Hz, while the operation wavelengths for the signal port is from 680 to 980 nm, a XYZ scanning stage with a customized handheld fiber bundle hosted on it and a research ultrasound platform for processing the imaging purposes. The detector of the ultrasound platform is a 128-element linear transducer array, which is in the middle of the bifurcated fiber bundles.

Two imaging systems were used in our experiments. The PA spectrums of nanoparticles’ solution, oil droplets with the diameter of 10 μm and microdroplets (the final products) were firstly investigated with Vevo LAZR-X (FUJIFILM VisualSonics, Inc, Toronto, ON, Canada), as shown in Fig. 9A. The ultrasound probe we selected was MX 550D, of which central frequency is 40 MHz and the bandwidth covers from 25 to 55 MHz. A built-in function ‘spectro’ was employed to do a B-scan PA imaging with the wavelengths swept from 680 to 970 nm and the step size was 5 nm. The purpose of these evaluations was to test the influence of the outer shell on the PA spectrum of nanoparticles. After the physical and optical properties of the microdroplets had been investigated, our previously developed handheld real-time photoacoustic system (HARP), which is shown in Fig. 9B, was applied for in vitro test and in vivo studies^26^. For HARP system, laser illumination was delivered by an optical parametric oscillator (OPO) (SpitLight EVO 200 OPO, InnoLas Laser GmbH, Krailling, Germany) with repetition rate of 100 Hz, while pumped by a diode-pumped neodymium-doped yttrium aluminum garnet (Nd:YAG) laser at 532 nm. Based on the absorption spectrum of CP NPs, 750 nm was chosen to trigger the PA effects. Light from the OPO was coupled into a customized fiber bundle (CeramOptec GmbH, Bonn, Germany) and delivered onto the target. The generated PA signals were then detected by a 128-element linear transducer array with central frequency of 18.5 MHz (L22-14v, Verasonics Inc., Kirkland, WA, USA) and processed by a 128-channel research US platform (Vantage 128, Verasonics Inc). To acquire volume images for in vivo studies, a customized XYZ 3-axis scanning stage (LS-110 with Hydra and Pollux controllers, Physik Instrumente (PI) GmbH & Co, Karlsruhe, Germany) was employed to perform the image scanning. We modified the code of a built-in function ‘Microvascular’, which provides the color Doppler images of small vessels, to evaluate the US contrast effect of the hydrogel-based microdroplets since assessment of the haemodynamic function is a typical application of US scanning^31^.

### Animal preparation

All animal experiments were approved by the Institutional Animal Care and Use Committee (IACUC) of the National University of Singapore (NUS) and performed in accordance with the guidelines and regulations of IACUC of NUS. The breast cancer cell line 4T1 was acquired from the American Type Culture Collection (ATCC). The cell line was maintained in RPMI-1640 culture medium with 10% fetal bovine serum and Penicillin–Streptomycin (10,000 U/ml). The 4T1 cells were then implanted into female NCr nude mice with age of 8–10 weeks under full anesthesia using mixture of ketamine 0.75 mg/ml and Medetomidine 0.1 mg/ml. Half million 4T1 cells suspended in 0.1 ml of PBS were subcutaneously injected in the right flank of mice. Tumor size was monitored using Vernier caliper. The subcutaneous tumor took approximately 7 days to grow to the size of 1 cm in diameter. Once it was ready, after anesthesia, the animal was mounted on the animal stage with abdomen facing upwards. Prepared agarose gel was put on top of the imaging area for coupling purposes.

## Supplementary information

Supplementary Information.

## Data Availability

The datasets generated during and/or analysed during the current study are available from the corresponding author on reasonable request.
