# Supplementary material for: Organic nanoparticle-doped microdroplets as dual-modality contrast agents for ultrasound microvascular flow and photoacoustic imaging
Source: Sci Rep. 2020 Oct 12;10:17009. doi: 10.1038/s41598-020-72795-w (PMC7550592; doi:10.1038/s41598-020-72795-w)
Supplement: Supplementary file 1 — Supplementary Information. [file 41598_2020_72795_MOESM1_ESM.docx]

**Organic nanoparticle-doped microdroplets as dual-modality contrast agents for ultrasound microvascular flow and photoacoustic imaging**

Yu Xu^1,2,*^, Guoyun Sun^1^, Eshu Middha^3^, Yu-Hang Liu^2^, Kim Chuan Chan^2^, Bin Liu^3^, Chia-Hung Chen^1,4,5^, Nitish V. Thakor^1,2,6,*^

^1^­Department of Biomedical Engineering, National University of Singapore, 4 Engineering Drive 3, 117583, Singapore, Singapore

^2^SINAPSE Laboratory, 28 Medical Drive, 117456, Singapore

^3^Department of Chemical and Bio-Molecular Engineering, National University of Singapore, 4 Engineering Drive 4, 117585, Singapore

^4^Institute for Health Innovation & Technology (iHealthtech), National University of Singapore (NUS), MD6, 14 Medical Drive #14-01, 117599, Singapore

^5^Department of Biomedical Engineering, City University of Hong Kong, 83 Tat Chee Avenue, Kowloon Tong, Hong Kong.

^6^Department of Biomedical Engineering, Johns Hopkins University, Baltimore, MD 21205, USA

Corresponding E-mail: [E0012410@u.nus.edu](mailto:E0012410@u.nus.edu), [thakornus@gmail.com](mailto:thakornus@gmail.com)

1. Microfluidic glass capillary mixer for synthesis of CP NPs


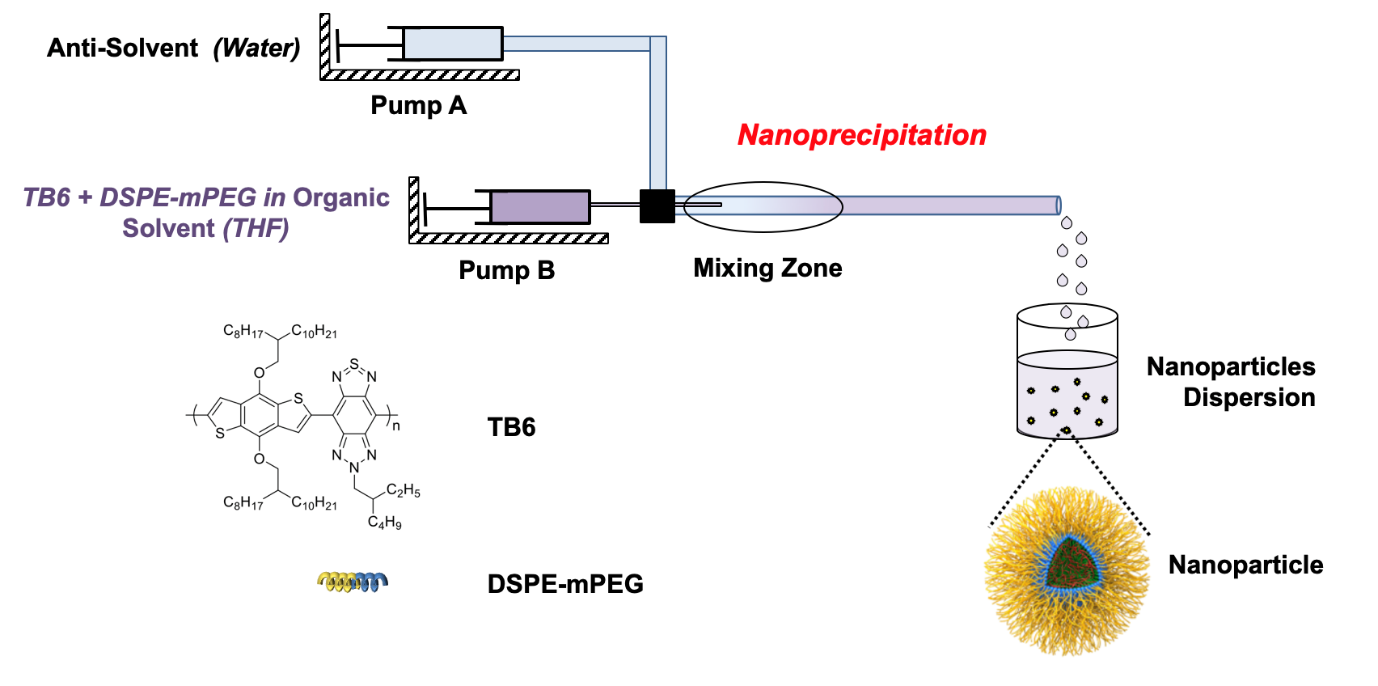


**Figure S1** Schematic of the CP NPs manufacturing process

1. Calculation of Reynolds number and flow velocity

The total flow rate in the system used was 34 ml/min. The relation between flow velocity (u) and volumetric flow rate (Q) is defined by Equation S1.

(S1)


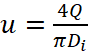

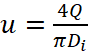


where D_i_ is the inner diameter of the pipe (1.5 mm)

Re in the system was varied on the basis of the total flow rate using the following Equation S2.

(S2)


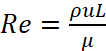

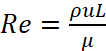


where μ represents the viscosity of fluid, ρ represents the density of the fluid and L is a characteristic linear dimension (m). For circular pipe, characteristic linear dimension is the same as the hydraulic diameter (inner diameter of pipe). Equation S3 represents the relation between Re and flow rate.

(S3)


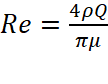

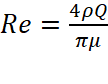


1. Microscopic image of the microdroplets and microdroplets in C-chip


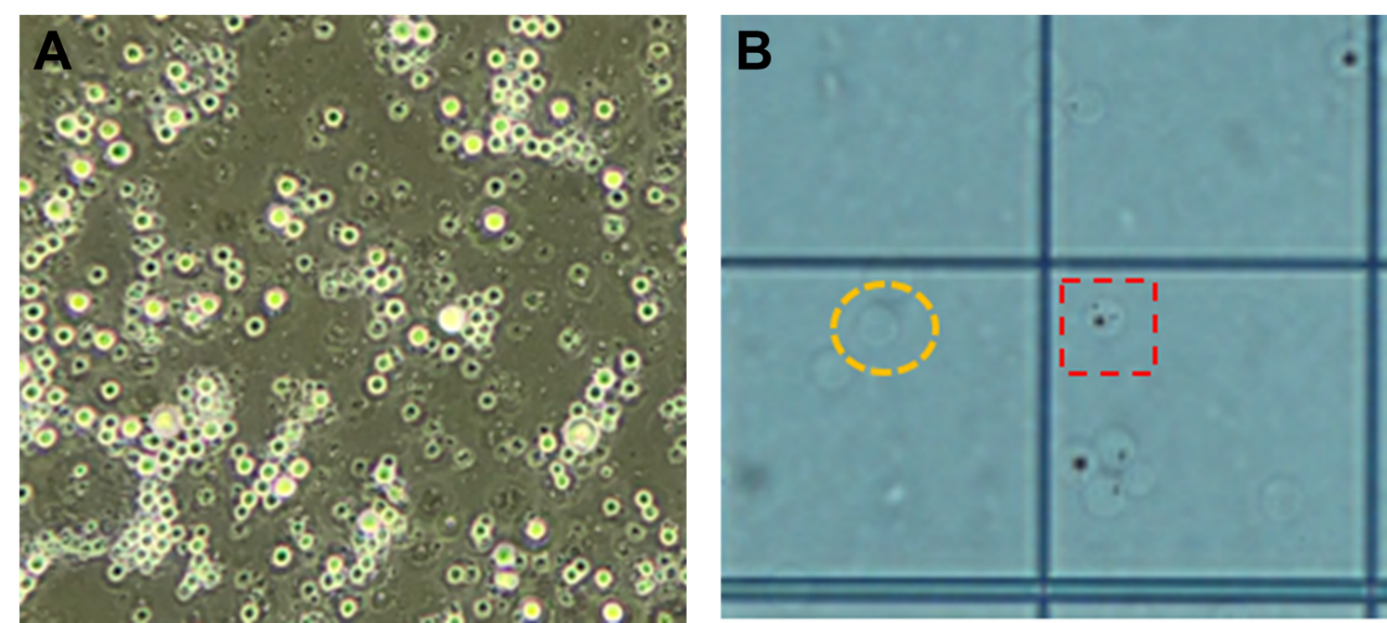


**Figure S2** (**A**) microscopic image of the final products under an inverted microscope (Eclipse Ti2, Nikon, Melville, U.S.A). Since the color of the CP NPs is dark green, the green dots inside the white or transparent circles indicate the CP NPs that have been successfully encapsulated with hydrogel. (**B**) Image taken by the camera while we were using C-chip to calculate the concentration and encapsulation efficiency of the microdroplets. Inside the dashed yellow circle is an empty microdroplets while the red dashed box has a successfully manufactured microdroplet inside.

1. Biodegradability verification


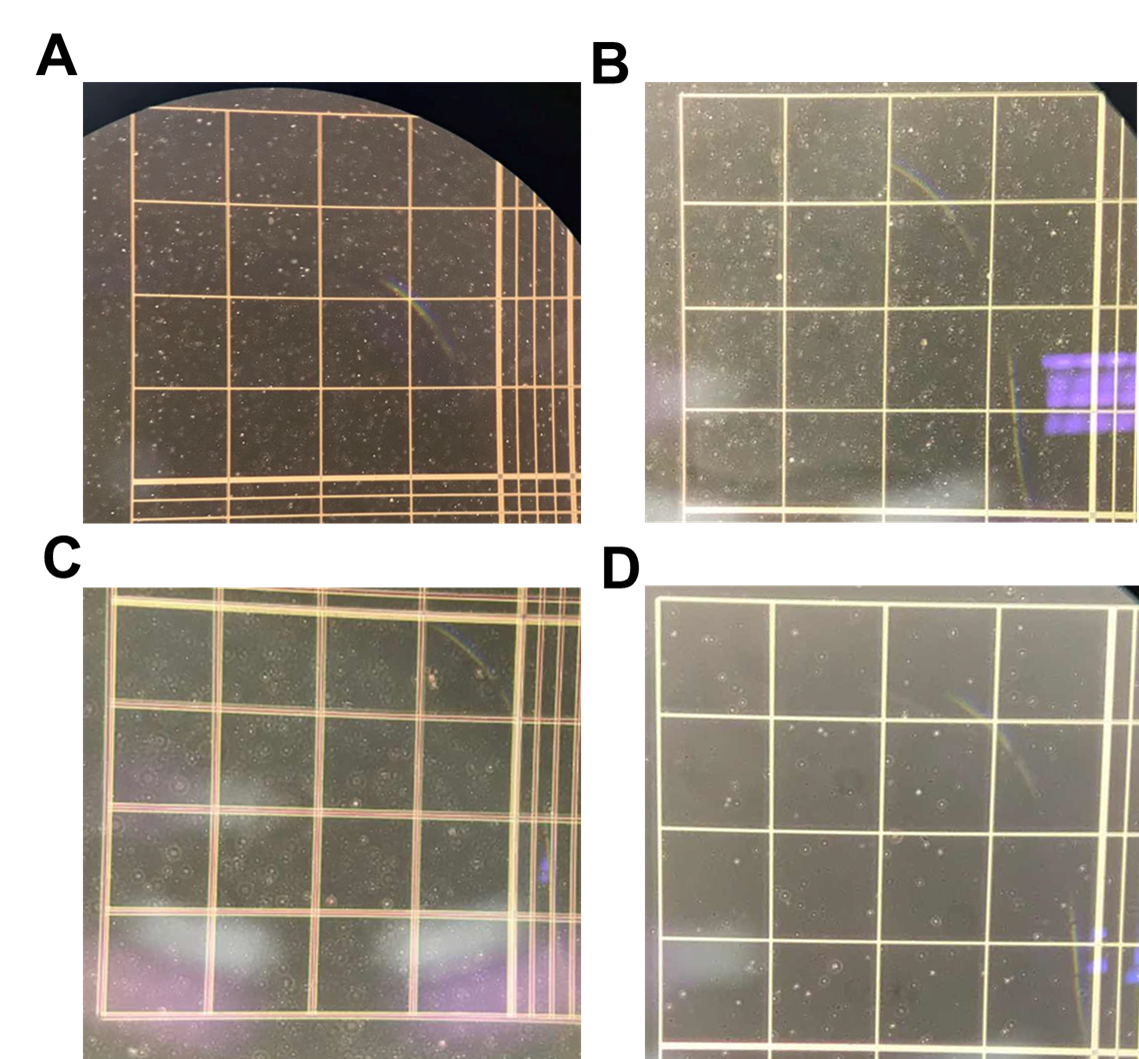


**Figure S3** The images of microdroplets in C-chip under an inverted microscope. (**A**) The image taken before water bath. (**B**) (**C**) and (**D**) are the images taken one and half hours, four and half hours and 24 hours after water bath.

The biodegradable property of the microdroplets was verified before the *in vitro* and *in vivo* experiments were conducted. 100 𝜇l microdroplets PBS solution was collected and put into a 2 ml tube. After changing the pH of the solution to 7.4, the concentration of the microdroplets was calculated using C-chip, which was around $4.8\times{10}^{6}$ /ml (*i.e.* starting point). After that, the tube was put into a 37 °C water bath (SWB series, Stuart, Cole-Parmer, Beacon Road, Stone, Staffordshire, ST15 OSA, UK) and samples were taken out at different time points to be observed and calculated the concentration. **Figure S3A** is the image taken before water bath. Subfigure **B**, **C** and **D** are the images taken after one and half hours, four and half hours and 24 hours after the water bath respectively. The images were taken using an inverted microscope (Eclipse Ti2, Nikon, Melville, U.S.A). From the images we can tell that after one and half hours in water bath, the microdroplets started swelling and in four and half hours after water bath, the swelling effects were most evident. Twenty-four hours later, the concentration was approximately $1.12\times{10}^{6}$ /ml, which was less than one fourth of the original concentration while even more microdroplets were swelling. Thus, our studies showed that the hydrogel based dual-modality contrast agents were biodegradable and could be degenerated in animal bodies.

1. Preparation of animal studies

We prepared both healthy and tumor induced nude mice for the *in vivo* imaging. Below is the example for the imaging of femoral vein in healthy animal. Before experiment, the microdroplets were diluted to $3.8\times{10}^{6}$ /ml and the pH value has been changed to 7.4 by adding NaOH solution. The prepared sample is shown in **Figure S4A**. After that, 2% agarose solution was heated up to prepare a transparent gel for PA wave coupling purpose (**Figure S4B**). Since the focal length of our linear transducer array and the fiber bundle are both 10 mm, the thickness of the gel we made was 10 mm. Lastly, the animal was anesthetized and was put on the animal stage (*i.e.* **Figure S4C**), the red dashed circle indicate the femoral vein we are going to visualize.


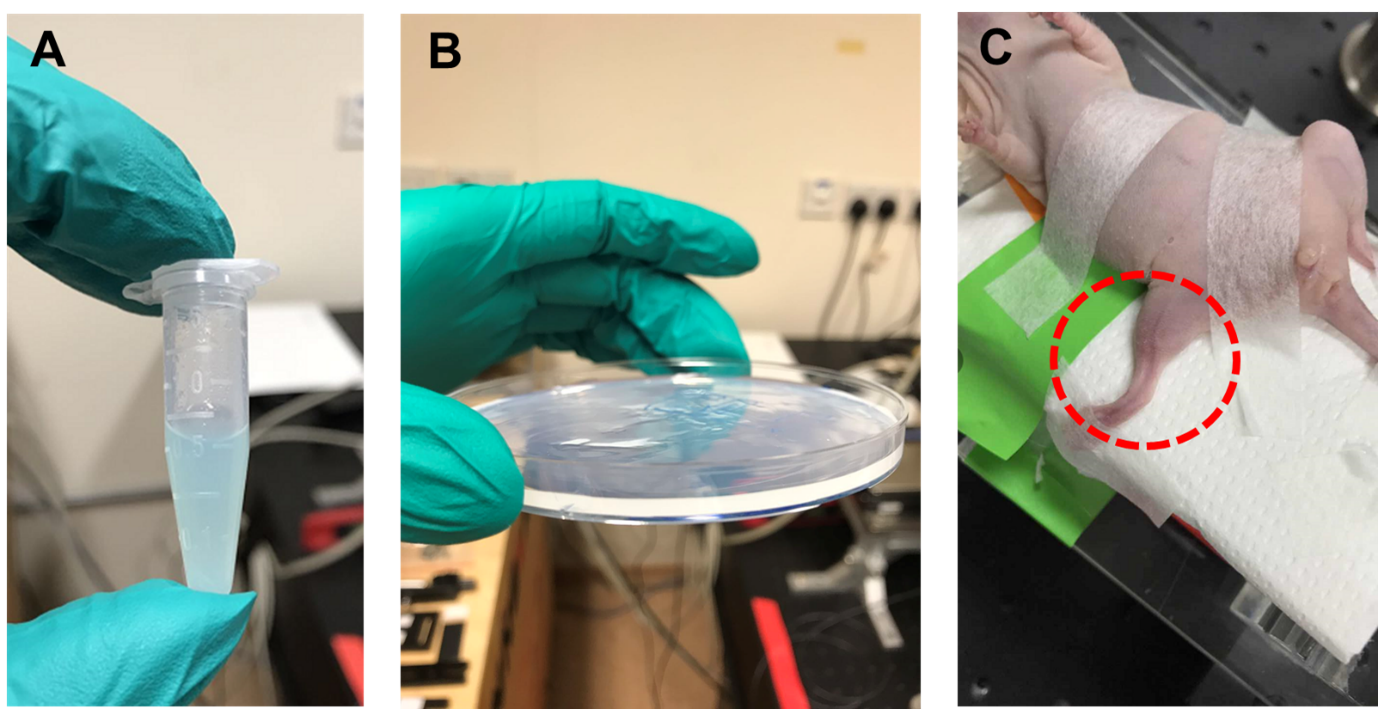


**Figure S4** (**A**) Prepared microdroplets for animal studies with concentration of $3.8\times{10}^{6}$ /ml and pH=7.4. (**B**) Prepared agarose gel for coupling purpose. (**C**) Anesthetized nude mouse on animal stage. The red dashed circle indicates the femoral vein that was imaged.
